# A Comparative Characterization of Physicochemical and Antioxidants Properties of Processed *Heterotrigona itama* Honey from Different Origins and Classification by Chemometrics Analysis

**DOI:** 10.3390/molecules24213898

**Published:** 2019-10-29

**Authors:** Sharina Shamsudin, Jinap Selamat, Maimunah Sanny, Shamsul Bahari A.R., Nuzul Noorahya Jambari, Alfi Khatib

**Affiliations:** 1 Faculty of Food Science and Technology, Universiti Putra Malaysia (UPM), Serdang 43400, Selangor, Malaysiamaimunah@edu.upm.my (M.S.); nuzuljambari@gmail.com (N.N.J.); 2 Food Science and Technology Research Centre, Malaysian Agricultural Research and Development Institute (MARDI), Persiaran MARDI-UPM, Serdang 43400, Selangor, Malaysia; 3 Food Safety and Food Integrity (FOSFI), Institute of Tropical Agriculture and Food Security, Universiti Putra Malaysia (UPM), Serdang 43400, Selangor, Malaysia; 4 School of Food Science and Technology, Universiti Malaysia Terengganu, Kuala Terengganu 21030, Malaysia; shamsul@umt.edu.my; 5 Pharmacognosy Research Group, Faculty of Pharmacy, International Islamic University Malaysia, Kuantan 25200, Pahang, Malaysia; alfikhatib1971@gmail.com

**Keywords:** stingless bee honey, physicochemical characteristics, antioxidants properties, free amino acids, partial least square-discriminant analysis (PLS-DA), chemometrics analysis

## Abstract

Stingless bee honey produced by *Heterotrigona itama* from different botanical origins was characterised and discriminated. Three types of stingless bee honey collected from acacia, gelam, and starfruit nectars were analyzed and compared with *Apis mellifera* honey. The results showed that stingless bee honey samples from the three different botanical origins were significantly different in terms of their moisture content, pH, free acidity, total soluble solids, colour characteristics, sugar content, amino acid content and antioxidant properties. Stingless bee honey was significantly different from *Apis mellifera* honey in terms of physicochemical and antioxidant properties. The amino acid content was further used in the chemometrics analysis to evaluate the role of amino acid in discriminating honey according to botanical origin. Partial least squares-discriminant analysis (PLS-DA) revealed that the stingless bee honey was completely distinguishable from *Apis mellifera* honey. Notably, a clear distinction between the stingless bee honey types was also observed. The specific amino acids involved in the distinction of honey were cysteine for acacia and gelam, phenylalanine and 3-hydroxyproline for starfruit, and proline for *Apis mellifera* honey. The results showed that all honey samples were successfully classified based on amino acid content.

## 1. Introduction

Stingless bee honey is a valuable product made by stingless bees. It has been reported to have higher nutritional and medicinal values compared to *Apis mellifera* honey [1]. However, the production of stingless bee honey is limited mainly because of the low quantities produced by stingless bees [2]. Nevertheless, stingless bee honey has been reported to be beneficial for human health due to the high antioxidant content [3]. It is also estimated that the price of stingless bee honey is much higher than *Apis mellifera* honey [4]. For instance, the market price of stingless bee honey can be as high as AU 50/kg [5]. Recently, there has been an increasing demand for pure and high-quality stingless bee honey as low-quality honey is usually produced with the addition of adulterants such as sweeteners (cane sugar, beet sugar, corn syrup, high fructose or maltose syrup) [6]. This practice is thought to alter its nutritional value and medicinal benefits. The quality of honey can also be characterised by its purity and source of origin. The purity of honey can be determined by its physicochemical properties (moisture content, pH, free acidity, total soluble solids, sugar content, colour characteristics and intensity, 5-hydroxymethylfurfural (5-HMF) content, and amino acid content), while the source of origin is influenced by several factors such as botanical, geographical and entomological origins [7]. Hence, the evaluation of physicochemical properties and authentication of botanical origins is vital to ascertain the quality and authenticity of honey. It is also evident that the quality and source of origin of honey may influence its market price and consumer acceptance, and, hence, it is important to determine these factors to guarantee the consumers’ safety and protect them against fraud.

Pollen analysis, traditionally known as the melissopalynalogical method, has been widely used to determine the botanical origin of honey and is performed through the identification of pollen constituents present in honey. This technique, however, is a very time-consuming and tedious process that relies on the availability of a comprehensive database of pollen grains and requires a well-trained analyst with a good knowledge of pollen morphology [8]. At present, other components in honey such as free amino acids [9], volatile compounds [10], protein content [11], carbohydrates content [12], and phenolic acids content [1] have been confirmed as markers for the identification of botanical and geographical origins of *Apis mellifera* honey.

In previous studies involving stingless bee honey, numerous parameters have been used to differentiate stingless bee honey types according to their botanical and entomological origins such as compositional features [13], physicochemical and antioxidant properties [14,15], chemical properties and mineral content [16], and a combination of sensorial, physicochemical and sugar content [17]. However, to date, there is no study performed on the use of amino acids as variables in classifying stingless bee honey according to their botanical origins.

Honey contains approximately 1.0 % (w/w) of amino acids derived mainly from the fluids and nectar secretions of the salivary glands and pharynx of honeybees. Pollen, however, has been reported as the main source of amino acids in honey [6]. There are many types of amino acids detected in honey such as alanine, asparagine, glutamine, histidine, glycine, arginine, valine, tyrosine, cysteine, lysine and others [18]. Therefore, these amino acids can be employed as a useful indicator to classify honey based on its botanical origin. Some amino acids have been identified as specific chemical markers for certain types of honey [9,19,20,21].

At present, the determination of botanical and geographical origins of honey is achieved using chemometric techniques to analyse complex data, extract useful information and simplify the analysis [20]. Additionally, support from modern statistical and quantitative data analysis techniques is required to obtain accurate and reliable results. By applying these techniques, the properties of honey and its corresponding constituents can be analysed, identified and classified. In this study, three types of stingless bee honey from acacia, gelam and starfruit nectars were investigated for their physicochemical properties and botanical origins, in which *Apis* honey was used for comparison. The aims of this study were to (1) assess the impact of botanical origins on the physicochemical properties and (2) affirm the botanical origin of the honey samples based on the amino acids content using chemometric techniques and, additionally, determine the amino acid markers that discriminate them.

## 2. Results and Discussion

### 2.1. Physicochemical Properties

The values for moisture content (before and after the drying process), pH, free acidity, total soluble solids (TSS), colour intensity, colour characteristics and 5-hydroxymethylfurfural (5-HMF) are shown in Table 1. Among all these parameters, moisture content, acidity, 5-HMF and sugars content have been stipulated by Codex Alimentarius (2001) [22] as the quality parameters for honey (*Apis mellifera*). According to Codex Alimentarius (2001), honey (*Apis mellifera*) must contain moisture content less than 20 g/100 g of honey, acidity value below than 50 meq/kg of honey and 5-HMF content, must less than 80mg/kg of honey. Based on the results obtained, the *Apis* honey sample met all the requirements. The moisture content, free acidity and 5-HMF content of the *Apis* honey sample were 14.67 g/100 of g, 39.22 meq/kg of honey and not detected (ND), respectively. This indicates that *Apis* honey was of a good quality, while all stingless bee honey samples met the requirements set by the Malaysian stingless bee honey standard [23]. According to the Malaysian stingless bee standard, raw stingless bee honey must contain moisture content less than 35 g/100 g of honey, pH less than 3.8 and 5-HMF less than 30 mg/kg of honey.

Overall, physicochemical results showed that all stingless bee honey had higher (21.52–25.49 g/100 g of honey) moisture content than *Apis* honey (14.67 g/100 g of honey). Stingless bee honey also had lower pH (3.00–3.27) and higher free acidity (107.50–246.25 meq/kg of honey) values as compared to *Apis* honey. In terms of total soluble solid (TSS), *Apis* honey showed higher (76.40 ^°^Brix) TSS than stingless bee honey. In addition to that, 5-HMF of all honey samples studied were low and some honeys such as acacia and *Apis* honey were free from 5-HMF. This indicates that all honey samples used were fresh.

Stingless bee honey is naturally high in moisture content. The moisture content values of all raw honey samples ranged from 14.67 to 25.49 g/100 g as shown in Table 1. *Apis* honey had the lowest moisture content of 14.67 g/100 g and was significantly lower compared to all the stingless bee honey samples, which ranged from 21.52 to 25.49 g/100 g. Stingless bee honey also showed significant differences in the moisture content among honey from different botanical origins, with acacia and gelam honey having the highest and lowest moisture content of 21.52 g/100 g and 25.49 g/100 g, respectively. Moisture content is a crucial parameter in the determination of honey quality. Based on the Codex Alimentarius (2001) [22], it is recommended that the moisture content of honey should not surpass 20 g/100 g. This is mainly because honey is vulnerable to fermentation and has low stability against microbes when the moisture content is higher than 20 g/100 g [24]. Generally, stingless bee honey owns an elevated moisture content than *Apis* honey [14]. This may be due to the different bee species and different preferences of bees towards the plant species used for honey production [25]. Issaro et al. [26] reported that honey from Thailand had lower levels of moisture content compared to our findings, in which the values were 15.73, 13.26 and 14.66 g/100 g for *Trigonalaeviceps Smith, Trigona* sp. and *Trigonapagdenis Schwarz* bee species, respectively. In contrast, honey from a Peruvian stingless bee species, *Partanoma epiphytophila,* possessed a higher moisture content of 45.80 g/100 g [27].

Owing to the high moisture content, stingless bee honey was subjected to the dehumidification process to reduce moisture content and avoid the honey sample from ferment. In this study, the moisture content of stingless bee honey samples obtained after the dehumidification process decreased by approximately 35.52% to 44.13%. The honey moisture content was lower than 20%, and therefore shown to comply with the Malaysian stingless bee honey standard for processed honey [23]. It is believed that honey moisture content levels lower than 20% can facilitate stingless bee honey preservation and prolong its shelf life.

In general, honey is naturally acidic [28]. Acidity has been reported to have a link with moisture content of the honey. Honey with high moisture content vulnerable to ferment and resulted in the high free acidity and low pH values. For instance, stingless bee honey samples had higher moisture content than *Apis* honey. Therefore, their pH was lower than *Apis* honey. The results in this study show that the pH and free acidity values obtained for the stingless bee honey samples varied between 3.00 and 3.27 and 107.50 to 246.25 meq/kg, respectively (Table 1). *Apis* honey, on the other hand, had a significantly higher pH and lower free acidity values compared to the stingless bee honey. Therefore, stingless bee honey was shown to be more acidic as opposed to *Apis* honey. Our findings are consistent with a previous study by Chuttong et al. [2] who also reported high free acidity values in stingless bee honey obtained from Thailand. Studies have shown that the organic acid content in honey is the main component responsible for the acidity of honey [29].

The total soluble solid has an association with moisture and sugar content in honey [25]. In general, honey with high total soluble solid possesses high sugar content and low moisture content. The total soluble solids of stingless bee honey from different botanical origins varied between 73.88 and 74.85 Brix (Table 1). The Brix value for *Apis* honey was significantly higher (76.40) than stingless bee honey.

All of the tested honey samples showed low 5-HMF content, ranging from not detected (ND) to 0.07 mg/kg as shown in Table 1. These findings indicate that the honey samples were of good quality as the values were below the maximum limit of 80 mg/kg as specified by the Codex Alimentarius (2001) [22]. 

The colour of honey is related to the minerals, phenolic compounds, and carotenoids present in honey [30]. Naturally, the colour of honey differs greatly, ranging from yellow to amber, dark amber and black in some cases. In this study, the colour characteristics and colour intensity of stingless bee honey varied between 1.25 and 46.45 mm Pfund, and 32.25 and 280.00 mAU, respectively (Table 1). According to the United States Department of Agriculture (USDA) [31] colour standards, starfruit, gelam and *Apis* honey types are classified as extra light amber, whereas acacia honey is classified as water white honey. *Apis* honey, on the other hand, showed significantly lower values for both parameters compared to gelam honey. The differences in colour characteristics and colour intensity were attributed to the different botanical sources used by bees to produce honey.

The sugar concentration profiles of stingless bee and *Apis* honey are shown in Table 2. The overall mean values of total sugar in stingless bee honey ranged from 70.89 to 73.96 g/100, in which no significant differences were observed between honey samples from different botanical origins. In contrast, *Apis* honey had the lowest total sugar content with a mean value of 68.80 g/100 g and was shown to be significantly lower compared to stingless bee honey. It is thought that the low total sugar content in *Apis* honey could be attributed to the difference in bee species.

The concentration of fructose and glucose in stingless bee honey varied from 15.27 to 29.06 g/100 g and 17.42 to 27.54 g/100 g, respectively. However, the concentration of fructose and glucose in *Apis* honey was 33.99 and 32.24 g/100 g, respectively, and significantly higher than stingless bee honey. In general, fructose is present in high concentrations in honey. However, in this study, only acacia and gelam honey exhibited higher fructose content compared to glucose, while starfruit had a lower fructose but higher glucose content. Previous studies by Tukshita et al. [32] Chuttong et al. [2] and Fuenmayor et al. [4] also found a lower fructose content in *Geniotrigona thoracica* (Borneo, Malaysia); *Tetrigona melanoleuca* (Thailand) and *Plebeia* spp. (Colombia), respectively. In addition, all the honey samples were statistically different in terms of their fructose and glucose contents depending on the type of botanical origin. Apart from fructose and glucose, maltose was also detected in starfruit honey with a concentration of 0.89 g/100 g. Based on several other studies from different countries, the maltose content found in our samples were much lower compared to the findings reported by Tukshita et al. [32], Chuttong et al. [2] and Oddo et al. [33], whereby the values ranged from 20.3 to 53.00 g/100 g.

Sugar composition is directly related to the nectar of blossoms gathered by bees [6]. The enzyme, invertase, is produced by bees to breakdown sucrose into fructose and glucose [34]. Thus, the sucrose content in honey acts as an indicator to determine its maturity and quality [6]. Mature and good quality honey should contain a sucrose content that is lower than 5 g/100 g [22]. The sucrose content detected in stingless bee honey samples varied between 17.36 and 37.32 g/100 g, while, for *Apis* honey, a lower value of 2.57 g/100 g was observed. All the stingless bee honey samples showed significant differences in terms of their sucrose content as well. These data indicate that the stingless bee honey samples evaluated in this study did not reach the maturity stage during the harvesting process due to the incomplete transformation of sucrose into glucose and fructose. Besides nectar, climate conditions and geographical regions can contribute to the different sugar compositions in honey [6].

### 2.2. Amino Acid Profile

**Figure 1:**
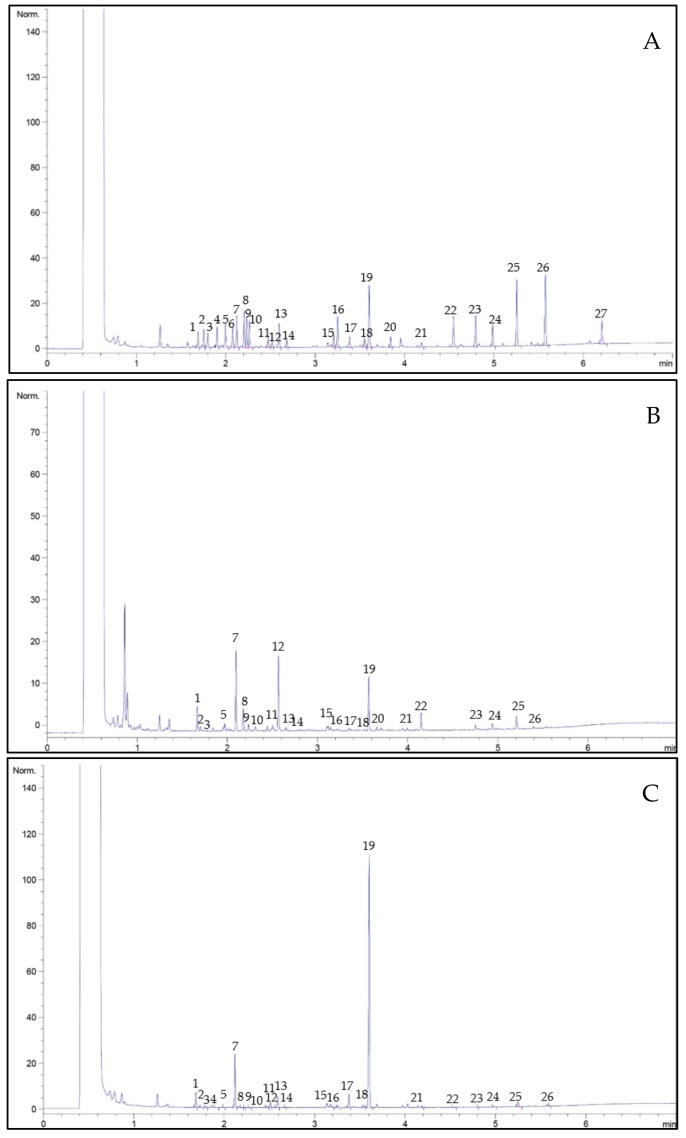
Representative gas chromatography flame-ionisation detection (GC-FID) chromatogram of amino acids standard mixture (100 umol/L) (**A**); *Apis* honey (**B**) and starfruit honey (**C**). For peak identification, 1 = Alanine; 2 = sarcosine; 3 = glycine; 4 = α-Aminobutyric acid; 5 = Valine; 6 = β-Aminoisobutyric acid; 7 = IS; 8 = Leucine; 9 = Allo-isoleucine; 10 = Isoleucine; 11 = Threonine; 12 = Serine; 13 = Proline; 14 = Asparagine; 15 = Aspartic acid; 16 = Methionine; 17 = 3-hydroxyproline; 18 = glutamic acid; 19 = Phenylalanine; 20 = α-Aminoadipic acid; 21 = Glutamine; 22 = Ornithine; 23 = Lysine; 24 = Histidine; 25 = Tyrosine; 26 = Tryptophan; 27 = Cysteine.

In this study, gas chromatography flame-ionisation detection (GC-FID) was successfully employed in the separation of amino acids in stingless bee and *Apis* honey samples. A typical GC-FID chromatogram of amino acids is shown in Figure 1. The limit of detection (LOD) and limit of quantitation (LOQ) for the amino acids in honey samples were 0.01–2.75 mg/kg and 0.02–8.33 mg/kg, respectively. The amino acids profile and concentration of stingless bee and *Apis* honey are shown in Table 3. All the amino acids analysed for the different honey types were detected, although some amino acids were found to be below the quantitation limit. For example, seven amino acids were detected in acacia, gelam and starfruit honey, while four amino acids were detected in *Apis* honey. The total amino acid content varied widely between 380.82 and 947.01 mg/kg. Among the stingless bee honey types, starfruit honey had the highest total content of amino acids at 947.01 mg/kg, while acacia honey had the lowest content at 380.82 mg/kg. Significant differences were also observed among the stingless bee honey samples in terms of the total amino acid content. For instance, when compared to *Apis* honey, starfruit honey samples exhibited a significantly higher value of total amino acid content, while acacia honey had a significantly lower value. Our results also showed a lower amount of total amino acid content compared to chaste honey (1572.90 mg/100 g) from China [20]. Nevertheless, starfruit honey showed a higher total amino acid content in comparison to buckwheat honey (633.50 mg/kg) from Poland and heather honey (655.10 mg/kg) from Estonia [21,35]. On the other hand, gelam and acacia honey had higher total amino acid contents than rape, willow, linden and dandelion honey [21]. These results suggest that the botanical origin can affect the amino acid composition in honey. At present, there is no data available on amino acid composition in stingless bee honey, and, hence, all comparisons were made based on the amino acid content in *Apis* honey.

Proline was significantly higher in *Apis* honey compared to stingless bee honey samples, while allo-isoleucine, cysteine, β-aminoisobutyric acid, and α-aminobutyric acid were not detected in *Apis* honey. Our findings were consistent with the results reported by Keskes et al. [9] and Qamer et al. [36], in which the authors also revealed a higher proline content in *Apis* honey. The differences in proline content may be attributed to the different species of bees involved in honey production. It has been reported that proline originates from the bees’ secretion and nectar used to make honey [37]. 

In acacia, starfruit and gelam honey, phenylalanine was the most abundant amino acid, ranging from 50.04 to 561.10 mg/kg, with starfruit honey having the highest value. Significant differences (*p* < 0.05) were also observed in the phenylalanine content among the stingless bee honey samples from different botanical origins. Sun et al. [20] reported higher amounts of phenylalanine in chaste honey from China, with a value of 1094.90 mg/100 g. The variation in phenylalanine content may be due to the different botanical and country origins of the honey. Many studies have also demonstrated and affirmed that the botanical origin and location can influence the types and concentration of amino acids present in honey [9,19,20]. The amino acids detected in stingless bee and *Apis* honey were used as variables in the chemometric analysis to classify the honey samples studied according to their botanical origins as well as to identify possible chemical markers that discriminate them.

### 2.3. Antioxidant Properties

Antioxidant capacity and activity of honey usually express as total phenolic content (TPC) and total flavonoids content (TFC) and free radical scavenging activity (IC_50_) and ferric reducing antioxidant power (FRAP) (Table 4). Antioxidant activity of honey mainly associated with the nectar source used by bees to make honey. In addition, phenolic compounds are derived from pollen and propolis constituents present in honey [38,39]. Botanical origin has been reported as the main factor that affect constituents and antioxidant activity of honey. Honey from different botanical origins have different antioxidant activity. The values of TPC detected in stingless bee honey were 61.47 mg GAE/100 g, 84.10 mg GAE/100 g and 114.49 mg GAE/100 g for acacia, starfruit and gelam honey, respectively (Table 4). Gelam and acacia honey had the highest and the lowest TPC values, respectively. Significant differences were also observed in TPC values among the different honey types, in which *Apis* honey had a significantly lower TPC value (29.05 mg GAE/100 g) compared to stingless bee honey. The variations in TPC value might be due to the different types of phenolic acids present in stingless bee and *Apis* honey [28]. This finding was consistent with the results reported previously in other studies [28,40].

In stingless bee honey samples, the flavonoids content (TFC) varied between 3.63 and 11.15 mg QAE/100 g as shown in Table 4, with significant differences (*p* < 0.05) observed between the different honey types. *Apis* honey contained significantly lower TFC values compared to the stingless bee honey types. It is thought that the variations observed in the TFC values could be due to the honey samples used in this study, which is comprised of different botanical origins, locations and bee species. Similar TFC values were observed in *M. beecheii* honey from Cuba (4.19 mg/100 g) [28] and *Trigona* spp. honey from Malaysia (4.46 to 7.91 mg QAE/100 g) [24]. On the other hand, Oliviera et al. [38] demonstrated higher TFC values in seven stingless bee honey samples from Bahia state, ranging from 30.24 to 279.73 mg QAE/100 g.

In general, honey with the lowest IC_50_ value possesses the highest antioxidant activity. The IC_50_ values found in all stingless bee honey samples analysed in this study ranged from 14.29 to 90.63 mg/mL (Table 4), while the IC_50_ for *Apis* honey was significantly higher at 202.15 mg/mL. Among the stingless bee honey types, gelam honey had the lowest IC_50_, followed by acacia and starfruit honey. These results suggest that gelam honey has the highest antioxidant activity compared to the other honey samples. All three types of stingless bee honey investigated in this study showed statistically different IC_50_ values. This observation may be due to the variations in phenolic content and types of phenolic compounds present in the honey samples [41]. Nevertheless, it has been reported that the phenolic content is correlated to antioxidant activity in honey [42,43,44]. Apart from phenolic acids, other compounds present in honey such as ascorbic acid, organic acids, amino acids, glucose oxidase, flavonoids and Maillard reaction products can also contribute to the antioxidant activity [45]. Higher IC_50_ values were detected in seven *Melipona* species honey samples from Bahia state, with values ranging from 25.39 to 51.44 mg/mL.

The FRAP values for the stingless bee honey investigated in this study ranged between 180.59 and 512.10 µmol Fe_2_SO_4_.7H_2_O/100 g (Table 4). As previously observed for the IC_50_ values, gelam honey exhibited the highest FRAP value and significant differences were observed in all the honey types. In comparison with other studies, the FRAP values obtained in this study were higher (38.54 µmol Fe_2_SO_4_.7H_2_O/100 g) than *Melipona beecheii* from Cuba and lower (668.88 µmol Fe_2_SO_4_.7H_2_O/100 g) than *Hypotrigona* sp. from Nigeria. The different types of phenolic compounds present in honey may influence the antioxidant activity of honey, owing to the difference in reducing potential [46]. *Apis* honey, however, had a significantly lower FRAP value than stingless bee honey due to the lower phenolic and flavonoids content in *Apis* honey compared to stingless bee honey.

### 2.4. Correlation Coefficients

A significant positive correlation coefficient of 0.981 was observed between the FRAP and TPC values as shown in Table 5, thereby indicating that the phenolic content may influence the reducing power activity of honey. However, no significant correlations were found between IC_50_ and TPC (−0.886) or TFC (−0.572) values, indicating that the antioxidant activity is not solely dependent on the phenolic and flavonoids content as other antioxidant compounds present in honey may also be involved. Previous studies by Ahmed et al. [47] and Idris et al. [41] also found similar results to those reported in this study. Likewise, there were no significant correlations between the colour of the honey and TPC (0.165) or TFC (−0.010) values. These results suggest that colour is not influenced by TPC or TFC in Malaysian stingless bee honey as previously reported in two studies involving honeybee honey (*Apis mellifera*) [44,48]. In these studies, the authors observed that dark honey contained high levels of phenolic and flavonoid content. 

### 2.5. Chemometric Analysis

#### 2.5.1. Partial Least Squares-Discriminant Analysis (PLS-DA)

PLS-DA is a supervised method commonly used to enhance the separation of groups through the identification of variables that focus on class separation [49]. Based on the PLS-DA results, all four principal components (PC) obtained in this study contributed to 97.77% of the variance, thereby indicating that PLS-DA provides a better classification than Principle Component Analysis (PCA) (data not shown). The PLS-DA model also had a goodness of fit value of 0.877 (R2X_cum_) and 0.978 (R2Y_cum_) and predictive value of 0.955 (Q2_cum_), with no outliers observed. 

**Figure 2:**
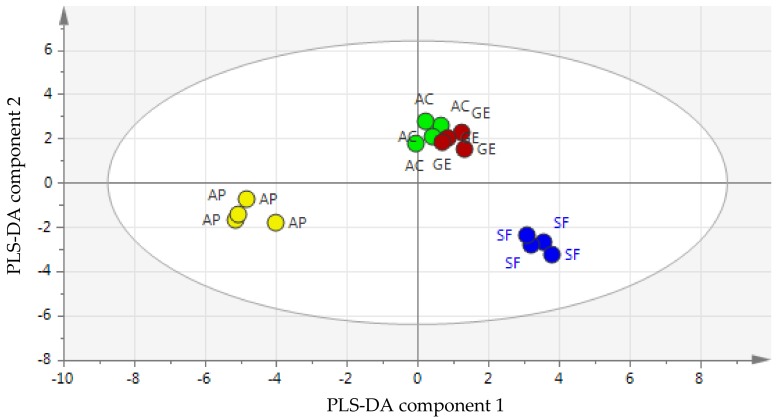
The Partial Least Squares-Discriminant Analysis (PLS-DA) score plot of amino acid data. Coloured circles are represented by AC = acacia honey (green); GE = gelam honey (red); SF = starfruit honey (blue) and AP = *Apis* honey (yellow).

The PLS-DA score plot represented in Figure 2 showed a clear separation between *Apis* honey and the stingless bee honey sample groups. These results demonstrated that amino acid content is an important discriminator of *Apis* honey from stingless bee honey. Based on PC1, starfruit, acacia, and gelam honey types were clearly distinguished from *Apis* honey, while the separation on PC2 showed that starfruit and *Apis* honey can be further differentiated from gelam and acacia honey. Thus, all the honey samples in this study were well-separated into four different groups, thereby indicating that amino acid content can be used as a discriminant to classify honey samples from different botanical origins.

**Figure 3:**
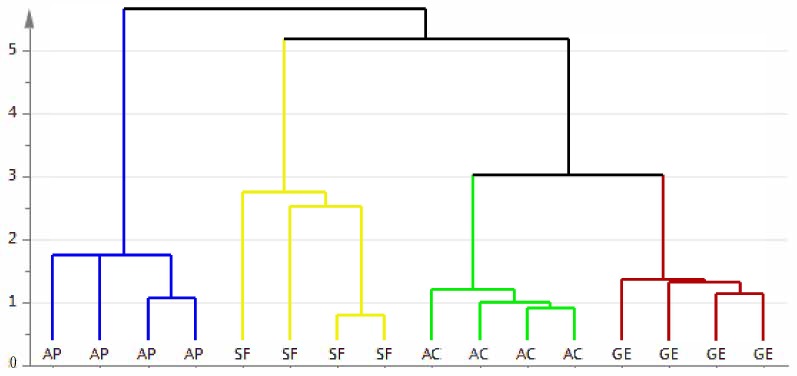
The dendrogram of the cluster analysis of stingless bee honey and *Apis mellifera* honey. AC = acacia honey; SF = starfruit honey; GE = gelam honey; AP = *Apis mellifera* honey.

Cluster analysis (CA) was further performed to evaluate the role of amino acids in classifying honey samples from different botanical origins (Figure 3) based on similarity. The results showed that honey from the same botanical origin are placed in the same group. There were no samples being assigned to the wrong group. This indicates that amino acids can be used as indicators to authenticate the botanical origin of honey.

**Figure 4:**
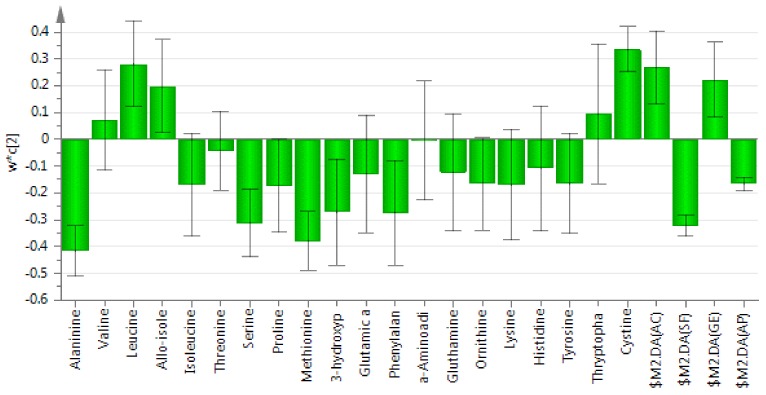
The Partial Least Squares-Discriminant Analysis (PLS-DA) loading column plot of amino acids in honey samples from different botanical origins. AC = acacia honey, SF = starfruit honey, GE = gelam honey and AP = *Apis* honey.

Figure 4 shows the PLS-DA loading column plot which highlights the variables responsible for the separation of honey samples. According to the loading column plot of PC2, three amino acids (leucine, allo-isoleucine, cysteine) contributed to the discrimination of acacia and gelam honey from starfruit and *Apis* honey. The starfruit and *Apis* honey samples are located on the negative side of the plot, with six amino acids (alanine, serine, proline, methionine, 3-hydroxyproline, phenylalanine) identified as discriminant variables. However, some variables were not considered as discriminants as their error bar exceeded zero [7]. These amino acids were identified as follows: valine, isoleucine, tryptophan, threonine, glutamic acid, α-aminoadipic acid, glutamine, ornithine, lysine, histidine, and tyrosine.

**Figure 5:**
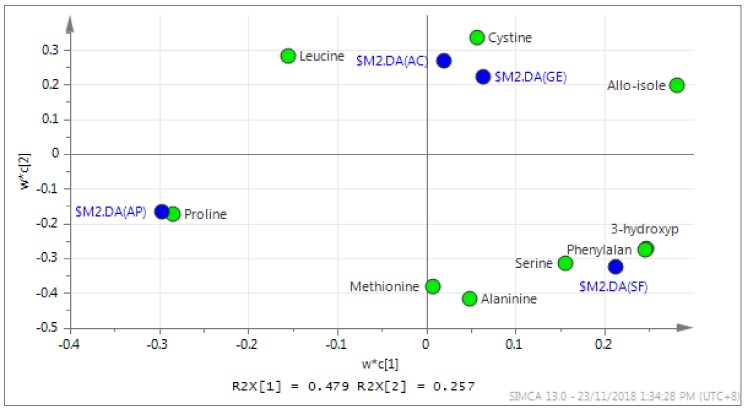
The score scatter plot of amino acids for all honey samples. The blue circle indicates the type of honey. AC = acacia honey, GE = gelam honey, SF = starfruit honey and AP = *Apis* honey.

The score scatter plot was performed to determine the most discriminatory amino acids responsible for grouping as well as identify potential markers for the honey samples (Figure 5). All amino acids that were not considered as discriminants were excluded from the score scatter plot. It was observed that some of the quantified amino acids were strongly associated with certain honey samples, in which amino acids that are located close to the honey sample have a strong discriminatory power compared to those located at a distance on the plot [50]. For instance, cysteine was a stronger discriminator compared to leucine and allo-isoleucine, and it can be used as a possible marker for acacia and gelam honey types. In addition, phenylalanine and 3-hydroxyproline were identified as possible markers for starfruit honey, while proline can be used to distinguish *Apis* honey from the stingless bee honey samples.

#### 2.5.2. Validation of the PLS-DA Model

**Figure 6:**
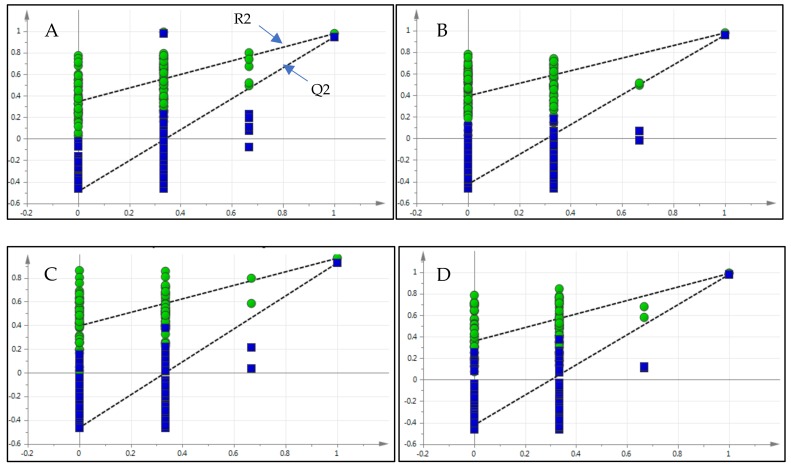
Graphs displaying the permutation tests for acacia (**A**); starfruit (**B**); gelam (**C**); and *Apis* (**D**) honeys.

To validate the PLS-DA model, permutation tests were performed for all the honey samples. The model is successfully validated when the R2-intercept and Q2-intercept do not exceed 0.3–0.4 and 0.05, respectively [49]. The permutation test results in Figure 6 exhibited Y-intercept and Q2-intercept pair values at 0.346 (R2) and –0.491 (Q2), 0.395 (R2) and –0.464 (Q2), 0.394 (R2) and –0.426 (Q2) and 0.346 (R2) and –0.491 (Q2) for acacia, gelam, starfruit and *Apis* honey types, respectively, thereby demonstrating that the PLS-DA classification model was successfully validated.

### 2.6. Potential Uses of Stingless Bee Honey

Honeybee and stingless bee honey have been reported to have ability to reduce food-borne toxicant formed during cooking meat and has a therapeutic effect due to their antioxidant activity. Previous studies have proved the role of honey in reducing heterocyclic amines (HCAs) in cooked meat [51,52]. In terms of therapeutic effect, honey has been used mainly to treat diseases related to the intestine and wounds [53]. For instance, applications of honey on wound can stimulate the healing process by stimulating tissue regeneration and reducing inflammation [53]. Apart from antioxidants, other honey constituents such as sugars (glucose and fructose), vitamins, minerals, proteins, hydrogen peroxide, flavonoids, phenolic acids, high acidity and high-water content may be involved in the wound healing process [54]. Currently, honey has been reported to prevent human diseases related to oxidative stress, such as cancer, cardiovascular disease, hypertension, diabetes mellitus and atherosclerosis [55]. Stingless bee honey has higher antioxidant activity than *Apis mellifera* honey [17]. Due to this reason, it is expected that stingless bee honey is always a better honey to treat wounds and all diseases related to the oxidative stress.

## 3. Materials and Methods

### 3.1. Standards and Chemicals

Sodium hydroxide (NaOH) was supplied by Sigma-Aldrich (St. Louis, MO, USA). The EZ:faast™ amino acid analysis kit was obtained from Phenomenex (Torrance, CA, USA). All reagents and standards for amino acids measured were provided within the kit. The water was filtered using an ELGA Pure Lab Classic system (ELGA, Woodridge, IL, USA). Methanol and acetonitrile were obtained from Merck (Darmstadt, Germany). All of the chemicals and solvents utilized were of analytical grade while of the High-performance Liquid Chromatography (HPLC) grade for HPLC analysis.

### 3.2. Geographical and Botanical Description of Honey Samples

Three natural honey samples produced by *Heterotrigona itama* were acquired directly from different beekeepers in Malaysia (Pahang, Terengganu and Malacca). The samples were gathered during flowering season between February and May 2017 to reduce variations from nectar of other flowers and guarantees the monofloral character of honey. Three honey samples from varying botanical origins, namely starfruit (*Averrhoa carambola L*), acacia (*Acacia mangium*) and gelam (*Meleleuca cajaputi* Powell) were used (Table 6). Acacia honey produced by honeybee honey (*Apis mellifera*) was used as a control in this study. Currently, there is no gelam and starfruit farms in Malaysia that produced both stingless bee and *Apis* honey. Only the acacia farm produced both stingless and *Apis* honey. Owing to this limitation, *Apis* honey from gelam and starfruit were not used in this study.

All honey samples were obtained from a local stingless bee farm. The identification of botanical origin was performed based on their geographical foraging area and floral availability where bee hives are located.

Gelam honey was collected from a stingless bee farm in Kuala Linggi Malacca, Malaysia. This farm was planted with three acres of gelam trees (*Meleleuca cajaputi* Powell) from *Myrtaceae* family and commonly known as kayu putih in Malaysia [56]. Honey samples were produced mainly from nectar of gelam flowers, while starfruit honey was gathered from a farm planted with two hectares of starfruit trees in Lanchang, Temerloh, Pahang, Malaysia. The major nectar collected by the bees is from the plant *Averrhoa carambola L*. from *Oxalidaceae* family [57,58]. Acacia honey was collected from a stingless bee farm located in the middle of the acacia forest in Sedili, Kota Tinggi, Johor, Malaysia. This farm consisting of acacia trees (*Acacia mangium*) that grow naturally in the forest with the land area is about two hectares. Acacia belongs to *Fabaceae* family and is called forest mangrove [59]. The bees collected the nectar mainly from *Acacia mangium* sap and flowers.

All samples were extracted from honey pots using an electric vacuum pump (Rocker 300, Kaohsiung, Taiwan). All stingless bee honey samples were submitted to the dehumidification process (40 °C) to reduce moisture content around 13–15 mg/100 g honey since the maximum limit is below than 20 mg/100 g honey in order to provide a better preservation of honey. Prior to dehumidification process, the water content of raw honey samples was evaluated. The processed honey samples were stored at 4 ± 2 °C in airtight plastic containers until further examination [60].

### 3.3. Physicochemical Analyses

#### 3.3.1. Moisture Content

The AOAC official method 969.38 [61] was referred to determine the moisture content of honey sample. Briefly, each honey sample was placed in vacuum oven at temperature of 60±2 °C with pressure at ≤50 mm Hg for 6 h. The moisture content was computed by dividing the weight of honey after drying with weight of honey before drying and time with 100%.

#### 3.3.2. Total Soluble Solid

A digital refractometer (ATAGO, Tokyo, Japan) was used to determine the total soluble solid of honey samples as described by Colucci et al. [62]. Approximately 0.3 mL of honey was spotted onto the glass prism of the refractometer and the measure was recorded at 25 °C in Brix.

#### 3.3.3. pH and Free Acidity

The AOAC official method 962.19 [61] was employed to determine pH and free acidity of honey samples. The pH and acidity were measured using a calibrated pH meter (calibrated at pH 4.00, 7.00 and 9.00 using buffer solutions). The pH was recorded after diluting a 10 g honey in 75 mL distilled water (pH 8.50). Then, 0.1 M NaOH was titrated into honey solution until the pH reached 8.50. The acidity was calculated based on differences in the volume of NaOH used in honey solution and distilled water. The results were expressed in milliequivalents (meq) of acid per kg of honey.

#### 3.3.4. Honey Colour Characteristics and Intensity

Colour characteristic [47] and colour intensity [63] of all honey samples were determined by using spectrophotometric analysis. The absorbance of diluted and filtered honey sample (1 g honey in 10 mL (w/v) distilled water) was measured using GENESYSTM 10S UV-Vis spectrophotometry from Thermo Fisher Scientific (Waltham, MA, USA) at 636 nm. Then, the absorbance data were recorded in Pfund value by utilizing this equation: mm Pfund = -38.7 + 371.39 × Abs [63]. For colour characteristics, the same diluted honey sample was measured for its absorbance at 450 nm and 720 nm. The distinction of the absorbance was reported in mAU.

#### 3.3.5. 5-hydroxymethylfurfural (5-HMF)

The 5-HMF content in the honey samples was measured using reversed-phase high-performance liquid chromatography (HPLC-RP) according to the approach established by Harmonised Methods of the International Honey Commission (HMIHC) [64]. The 5-HMF solution was extracted from honey samples according to Gokmen and Acer [65]. Subsequently, the 20 µL of 5-HMF solution was injected into a reversed-phase high-performance liquid chromatography (WATERS, Milford, MA, USA) coupled with a photodiode array detector (WATERS 2996, Milford, MA, USA). The separation of 5-HMF was facilitated by a mixture of mobile phase, water (90%) and methanol (10%) using a reversed-phase column, Luna^®^ C18(2) 100 Å (250 mm × 4.6 mm × 5 µm) from Phenomenex Inc. (Torrance, CA, USA). A continual flow rate of 1.0 mL/min was applied for 30 min. A 5-HMF standard was used to determine the 5-HMF in the samples by comparing the corresponding peak of the samples and 5-HMF standard. A standard curve of five different concentrations of 5-HMF was constructed to compute the amount of 5-HMF in honey samples and express in mg/kg honey.

#### 3.3.6. Sugar Profile

The detection and quantitation of sugar in the honey samples were performed using high-performance liquid chromatography integrated with a refractive index detector (HPLC-RI) as established by Harmonised Methods of the International Honey Commission (HMIHC) [64] with some modifications. Separation of sugars in the honey sample was carried out using an amino column Luna® NH2 100 Å (250 mm × 4.6 mm × 5 µm) from Phenomenex Inc. (Torrance, CA, USA) with acetonitrile:water (80:20) as a mobile phase. The temperature of column was set at 40 °C to obtain an efficient separation. A volume of 10 µL honey solution (0.5 g of honey in 10 mL of distilled water) and the flow rate of 1 mL/min was used. The analysis was observed for 20 min. Each sugar was distinguished by comparing the retention time of genuine standard. An equation from the calibration curve of each sugar standard was used to calculate sugar concentrations and expressed in gram sugar per 100 g of honey.

#### 3.3.7. Amino Acid Profile

The amino acids profile was analysed using gas chromatography with flame ionization detection as reported by Nozal et al. [19] and Mustafa et al. [66]. All honey samples were subjected to derivatization process prior to gas chromatography analysis. About 100 mg honey was dissolved with 500 uL distilled water and the solution was homogenised using a vortex mixer. The Phenomenex EZ:faast™ amino acid analysis kit (Phenomenex, Torrance, CA, USA) was used to extract and derivatize honey solution (30 uL) prior to GC-FID analysis. The separation of derivatised amino acids were performed using Agilent 7890A GC-FID (Agilent Technologies, Wilmington, DE, USA). The column used was Zebron ZB-AAA capillary GC column (10 m × 0.25 mm id, Phenomenex, Torrance, CA, USA). The column oven temperature program as follows: 110 to 320 °C at 32 °C/min. The FID detector temperature was 320 °C and 1 μL of each sample was injected at an injection temperature of 250 °C and a split level of 1:15. The carrier gas was helium at a pressure of 3 kPa/min (a flow rate of 1.5 ml/min). Identification of chromatographic peaks were performed by comparing the retention times of the standards and the samples components. Quantification was carried out based on the standard curve obtained from five amino acids standards mix with known concentrations (50–400 µmol/L). The limit of detection (LOD) and limit of quantitation (LOQ) values were computed utilizing these formulas, 3.3*standard deviation of blank response/slope and 10*standard deviation of blank response/slope separately [67]. The LOD and LOQ of amino acids were varied from 0.01 to 2.36 mg/kg of honey and 0.02 to 7.15 mg/kg of honey, respectively.

### 3.4. Analyses of Antioxidant Properties

#### 3.4.1. Total Phenolic Content (TPC)

The Folin–Ciocalteu method [68] with slight modification was used to estimate the concentration of total phenolic content (TPC). Initially, the honey solution was prepared by mixing 100 mg of honey (dry basis) with 3 mL methanol. Then, approximately 200 µL of honey solution was added with 1000 µL of Folin–Ciocalteu (FC) reagent (dilution ratio of FC reagent, 1:10 v/v), and incubated for 6 min in the dark. Afterwards, a 7.5% sodium carbonate solution (800 µL) was added, shaken and incubated in the dark for 2 h. Later, the absorbance was read at 740 nm against a methanol blank (GENESYSTM 10S UV-Vis spectrophotometry, Waltham, MA, USA). The TPC of the honey samples was computed according to the equation gained from a calibration curve of a standard solutions gallic acid and expressed in gallic acid equivalent (GAE/100 g honey).

#### 3.4.2. Total Flavonoids Content (TFC)

The spectrophotometric approach was employed to estimate the total flavonoids content (TFC) in honey samples while quercetin was used as a reference [69]. Briefly, a mixture of 1 mL of honey solution (500 mg honey (dry basis) in 2 mL methanol) and 0.3 mL of 5% NaNO_2_ solution was mixed for 5 min and 0.3 mL of 10% AlCl_3_ was added. The mixture was stirred for 6 min and the solution was neutralized by adding 2 mL of 1 M NaOH. The absorbance of each sample was measured at 510 nm against a methanol blank by utilizing a UV-VIS spectrophotometer (GENESYSTM 10S UV-Vis spectrophotometry) from Thermo Fisher Scientific (Waltham, MA, USA). The TFC was computed according to the equation obtained from a calibration curve of a standard quercetin acid solutions and expressed in mg quercetin acid equivalent (QAE)/100 g of honey.

#### 3.4.3. Free Radical Scavenging Activity (DPPH Assay)

The DPPH assay was used to estimate the radical scavenging activity of honey sample based on the method reported by Meda et al. [70] with a slight alteration. The radical scavenging activity was expressed as IC_50_ (concentration of honey solution required to mitigate the initial concentration of DPPH by 50%). Shortly, five serials of methanolic honey dilution were prepared. Later, 1.5 mL (0.02 mg/mL) of a methanolic solution of DPPH was added to the 0.75 mL methanolic honey dilution and mixed thoroughly. The mixtures were maintained in the dark for 15 min at room temperature. The absorbance of each solution was read at 517 nm against a methanol blank using a UV-VIS spectrophotometer (GENESYSTM 10S UV-Vis spectrophotometry) from Thermo Fisher Scientific (Waltham, MA, USA). The radical scavenging activity was calculated as the percentage of radical scavenging activity (RSA) using the following formula: Radical scavenging activity = ((A_DPPH_ − As) / A_DPPH_) × 100, where As is the absorbance of the sample solution and A_DPPH_ is the absorbance of the DPPH solution.

#### 3.4.4. Ferric Reducing Antioxidant Power (FRAP Assay)

FRAP assay was carried out to evaluate the ability of antioxidants in honey sample to reduce ferric ion (Fe^3+^) to ferrous ion (Fe^2+^) according to Khalil et al. [71] with some modifications. The FRAP value is expressed as micromoles of ferrous equivalent (µM Fe (II)) per kilogram of honey. Initially, 200 µL of honey solution (100 mg of honey (dry basis) in 11 mL methanol) was mixed with 1.5 mL of FRAP reagent. After incubation at 37 °C in a water bath for 4 min, the absorbance was measured at 593 nm against a methanol blank using a UV-VIS spectrophotometer (GENESYSTM 10S UV-Vis spectrophotometry) from Thermo Fisher Scientific (Waltham, MA, USA). Fresh FRAP reagent was prepared by mixing 10 mL of 300 mM/L acetate buffer (pH 3.6) with 1 mL of 10 mmol 2,4,6-tris(1-pyridyl)-1,3,5-triazine (TPTZ) and 1 mL of 40 mM/L HCl containing 20 mM ferric chloride (FeCl_3_ 6H_2_O). Then, the FRAP reagent solution was pre-warmed at 37 °C prior to use. The quantification of antioxidant activity was done by constructing a calibration curve of standard ferrous sulphate (FeSO_4_ 7H_2_O) solutions against the concentration of honey solution.

### 3.5. Chemometrics Analysis

All four honey samples were subjected to chemometrics analysis. In chemometrics analysis, the amino acid profile was used as variables. A total of 20 amino acids from each honey sample were selected by the SIMCA software (version, Manufacturer, City, US State abbrev. if applicable, Country) and used to develop a partial least squares discriminant analysis (PLS-DA) model. The cluster analysis (CA), score plot and the score scatter plot were performed to discriminate honey samples according to their botanical origin. Loading column plot was performed to determine the correlation between variables and sample. The quality of the model was explained by goodness of fit (R2X_cum_ and R2Y_cum_) and predictive values (Q2_cum_). The model was further validated with the permutation test [72,73].

### 3.6. Statistical Analysis

All experiments were conducted in four replicates and the results were presented as mean ± standard deviation. The difference between samples was analysed using one-way analysis of variance (ANOVA) at *p* < 0.05 using MINITAB software Version 17.0 (Manufacturer, Sydney, NSW, Australia). A correlation test was performed using Pearson’s correlation at *p* < 0.05. SIMCA software Version 13.0 (MKS Data Analytics Solutions, Umeå, Sweden) was used to evaluate the relationship between amino acids and honey classification based on their botanical origin. 

## 4. Conclusions

The physicochemical and antioxidant properties of stingless bee honey from acacia, starfruit, and gelam produced by *Heterotrigona itama* bees were characterized. The findings revealed that the physicochemical properties of the honey samples investigated were highly dependent on their botanical origins. The results also showed that the physicochemical properties and antioxidant activity of stingless bee honey were significantly different compared to *Apis mellifera* honey (honeybee honey). Moreover, the PLS-DA evaluation indicated that stingless bee honey was clearly distinguishable from *Apis mellifera* honey, and within the stingless bee honey types, based on the amino acid profile. The amino acid profile coupled with chemometrics analysis has successfully classified stingless bee honey and proved the possibility of using chemometrics analysis to classify and identify biomarkers in stingless bee honey. In addition, stingless bee honey was shown to have a high antioxidant activity and therefore it is good for human health. 

## Figures and Tables

**Table 1:** Physicochemical characteristics of honey samples from different botanical origins.

Parameters	Unit	Botanical Origins of Honey			*Apis*
		Acacia	Starfruit	Gelam	*mellifera*
Moisture content *	g/100 g	21.52 ± 0.66 ^c^	24.24 ± 0.19 ^b^	25.49 ± 0.45 ^a^	14.67 ± 0.11 ^d^
Moisture content **	g/100 g	13.86 ± 0.38 ^b^	15.63 ± 0.41 ^a^	14.24 ± 0.65 ^b^	NA
pH	pH	3.27 ± 0.03 ^b^	3.00 ± 0.03 ^d^	3.18 ± 0.03 ^c^	3.56 ± 0.02 ^a^
Free acidity	meq/kg honey	107.50 ± 6.45 ^c^	246.25 ± 9.46 ^a^	176.25 ± 9.46 ^b^	39.22 ± 1.50 ^d^
TSS	^°^Brix	74.65 ± 0.39 ^bc^	73.88 ± 0.34 ^c^	74.85 ± 0.73 ^b^	76.40 ± 0.54 ^a^
Colour	mm Pfund	1.25 ± 0.10 ^d^	36.85 ± 2.00 ^c^	46.45 ± 2.45 ^a^	40.70 ± 0.53 ^b^
Colour intensity	mAU	32.25 ± 3.59 ^d^	122.50 ± 9.57 ^c^	280.00 ± 18.26 ^a^	251.00 ± 2.16 ^b^
5-HMF	mg/kg honey	ND ^b^	0.07 ± 0.06 ^a^	0.05 ± 0.02 ^a^	ND ^b^

* Before dehumidification process; ** After dehumidification process; TSS = Total soluble solid; 5-HMF = 5-Hydroxymethylfurfural; NA = Not available;.ND = Not detected. Mean values in the same row with distinct superscript letters indicate a significant difference at *p* < 0.05.

**Table 2:** The concentration of sugar (g/100 g) in honey samples from different botanical origins.

Parameters	Botanical Origin of Honey			*Apis*
	Acacia	Starfruit	Gelam	*mellifera*
Fructose	22.05 ± 0.85 ^c^	15.27 ± 0.51 ^d^	29.06 ± 1.52 ^b^	33.99 ± 0.31 ^a^
Glucose	21.17 ± 1.50 ^c^	17.42 ± 0.82 ^d^	27.54 ± 1.98 ^b^	32.24 ± 0.38 ^a^
Sucrose	28.44 ± 0.89 ^b^	37.32 ± 1.14 ^a^	17.36 ± 1.09 ^c^	2.57 ± 0.24 ^d^
Maltose	ND ^b^	0.89 ± 0.43 ^a^	ND ^b^	ND ^b^
Total sugar	71.65 ± 2.04 ^ab^	70.89 ± 0.80 ^bc^	73.96 ± 2.74 ^a^	68.80 ± 0.72 ^c^

ND = not detected. Mean values in the same row with distinct superscript letters indicate a significant difference at *p* < 0.05.

**Table 3:** Amino acids profile and concentration (mg/kg) in honey samples from different botanical origins.

Amino Acids	Botanical Origin of Honey			
	Acacia	Starfruit	Gelam	*Apis mellifera*
Alanine	35.04 ± 2.03 ^c^	46.00 ± 2.06 ^a^	35.96 ± 2.14 ^c^	41.34 ± 0.34 ^b^
Sarcosine	<LOQ	<LOQ	<LOQ	<LOQ
Glycine	<LOQ	<LOQ	<LOQ	<LOQ
α-Aminobutyric acid	<LOQ	<LOQ	<LOQ	ND
Valine	1.24 ± 0.77 ^b^	<LOQ ^b^	5.07 ± 1.65 ^a^	6.06 ± 1.92 ^a^
β-Aminoisobutyric acid	<LOQ	ND	<LOQ	ND
Leucine	24.82 ± 0.64 ^c^	7.55 ± 0.20 ^d^	44.50 ± 4.44 ^a^	32.91 ± 0.26 ^b^
Allo-isoleucine	7.03 ± 1.31 ^a^	6.19 ± 0.13 ^a^	6.38 ± 0.03 ^a^	ND ^b^
Isoleucine	<LOQ ^b^	<LOQ ^b^	<LOQ ^b^	7.06 ± 0.84 ^a^
Threonine	4.75 ± 3.67 ^b^	3.85 ± 1.32 ^b^	7.07 ± 3.21 ^ab^	12.08 ± 4.25 ^a^
Serine	13.60 ± 2.46 ^b^	37.75 ± 5.14 ^a^	19.07 ± 0.83 ^b^	19.21 ± 11.45 ^b^
Proline	16.23 ± 5.68 ^d^	33.01 ± 6.20 ^c^	48.91 ± 1.44 ^b^	145.9 ± 3.39 ^a^
Asparagine	<LOQ	<LOQ	<LOQ	<LOQ
Aspartic acid	<LOQ	<LOQ	<LOQ	<LOQ
Methionine	16.71 ± 0.21 ^c^	22.62 ± 0.56 ^a^	19.49 ± 0.19 ^b^	21.40 ± 2.14 ^a^
3-hydroxyproline	4.83 ± 3.01 ^c^	75.53 ± 5.47 ^a^	29.63 ± 2.28 ^b^	6.34 ± 0.77 ^c^
Glutamic acid	29.02 ± 4.27 ^b^	37.29 ± 4.15 ^a^	29.77 ± 2.31 ^b^	23.68 ± 0.59 ^c^
Phenylalanine	50.04 ± 4.76 ^c^	561.10 ± 37.59 ^a^	237.37 ± 13.73 ^b^	68.51 ± 0.35 ^c^
α-Aminoadipic acid	4.96 ± 1.00 ^ab^	ND ^b^	ND ^b^	7.69 ± 5.13 ^a^
Glutamine	42.15 ± 2.73 ^b^	24.75 ± 4.11 ^c^	29.32 ± 1.19 ^c^	134.09 ± 10.81 ^a^
Ornithine	11.26 ± 0.32 ^ab^	12.15 ± 0.87 ^a^	11.13 ± 0.50 ^b^	10.93 ± 0.59 ^b^
Lysine	15.46 ± 1.04 ^b^	15.17 ± 0.44 ^b^	14.67 ± 0.04 ^b^	21.94 ± 0.68 ^a^
Histidine	23.76 ± 4.09 ^b^	20.27 ± 2.08 ^bc^	17.82 ± 0.07 ^c^	30.03 ± 1.00 ^a^
Tyrosine	24.92 ± 0.84 ^b^	23.31 ± 2.63 ^b^	19.57 ± 1.97 ^c^	29.63 ± 0.66 ^a^
Tryptophan	20.32 ± 1.25 ^a^	20.46 ± 3.16 ^a^	19.05 ± 0.38 ^a^	20.58 ± 2.27 ^a^
Cysteine	34.67 ± 2.09 ^a^	ND ^b^	33.01 ± 2.62 ^a^	ND ^b^
TOTAL	380.82 ± 19.36 ^c^	947.01 ± 48.42 ^a^	627.78 ± 21.20 ^b^	639.47 ± 13.49 ^b^

<LOQ = below limit of quantitation; ND = not detected; Number of samples used for amino acids analysis; *n* = 4. Mean values in the same row with distinct superscript letters indicate a significant difference at *p* < 0.05.

**Table 4:** Antioxidant properties of honey samples from different botanical origins.

Parameters	Unit	Botanical Origin of Honey			
		Acacia	Starfruit	Gelam	*Apis mellifera*
**Total phenolic contents (TPC)**	mg GAE/100 g honey	61.47 ± 3.34 ^c^	84.10 ± 5.33 ^b^	114.49 ± 7.31a	29.05 ± 1.58 ^d^
Total flavonoids content (TFC)	mg QAE/100 g honey	3.63 ± 0.26 ^c^	11.15 ± 0.55 ^a^	8.41 ± 0.36 ^b^	1.57 ± 0.24 ^d^
Free radical scavenging activity (IC_50_)	mg/mL	58.35 ± 2.45 ^c^	90.63 ± 5.50 ^b^	14.29 ± 0.53 ^d^	202.15 ± 1.60 ^a^
Ferric reducing antioxidant power (FRAP)	µmol Fe_2_SO_4_.7H_2_O/100 g honey	180.59 ± 10.48 ^b^	263.90 ± 22.1 ^c^	512.10 ± 47.4 ^a^	40.22 ± 1.84 ^d^

GAE = Gallic acid equivalent; QAE = Quercetin acid equivalent; IC_50_ = concentration of honey solution required to mitigate the initial concentration of DPPH by 50%. Mean values in the same row with distinct superscript letters indicate a significant difference at *p* < 0.05.

**Table 5:** Correlation coefficients (r) between antioxidants properties and colour intensity of honey.

Parameters	TPC	TFC	IC_50_	FRAP	Colour Intensity
TPC	1.000				
TFC	0.802	1.000			
IC_50_	−0.886	−0.572	1.000		
FRAP	0.981 *	0.691	−0.863	1.000	
Colour intensity	0.165	−0.010	0.182	0.303	1.000

TPC = Total phenolic contents, TFC = Total flavonoids content, IC_50_ = Free radical scavenging activity, FRAP = Ferric reducing antioxidant power. (r) near to +1 or −1 indicates strong relationship and near to 0 indicates weak/no relationship. *Correlation is significant at *p* < 0.05.

**Table 6:** Stingless bee honey samples from varying botanical origins.

Sample	Scientific Name	Geographical Origin	Bee Species
Gelam Acacia Starfruit	*Meleleuca cajaputi* Powell *Acacia mangium* *Averrhoa carambola L*	Malacca Johor Pahang	*Heterotrigona itama*
Acacia	*Acacia mangium*	Johor	*Apis mellifera*
